# Does an instrumented treadmill correctly measure the ground reaction forces?

**DOI:** 10.1242/bio.20136379

**Published:** 2013-11-06

**Authors:** Patrick A. Willems, Thierry P. Gosseye

**Affiliations:** 1 Laboratoire de Physiologie et Biomécanique de la Locomotion, Institute of NeuroScience, Université Catholique de Louvain, B-1348 Louvain-la-Neuve, Belgium; 2 Arsalis SPRL, 6 Chemin du Moulin Delay, B-1473, Glabais, Belgium

**Keywords:** Locomotion, Instrumented treadmill, Ground reaction forces, Force transducers

## Abstract

Since the 1990s, treadmills have been equipped with multi-axis force transducers to measure the three components of the ground reaction forces during walking and running. These measurements are correctly performed if the whole treadmill (including the motor) is mounted on the transducers. In this case, the acceleration of the treadmill centre of mass relative to the reference frame of the laboratory is nil. The external forces exerted on one side of the treadmill are thus equal in magnitude and opposite in direction to the external forces exerted on the other side. However, uncertainty exists about the accuracy of these measures: due to friction between the belt and the tread-surface, due to the motor pulling the belt, some believe that it is not possible to correctly measure the horizontal components of the forces exerted by the feet on the belt. Here, we propose a simple model of an instrumented treadmill and we demonstrate (1) that the forces exerted by the subject moving on the upper part of the treadmill are accurately transmitted to the transducers placed under it and (2) that all internal forces – including friction – between the parts of the treadmill are cancelling each other.

## Introduction

For many years, motorized treadmills have been used to mimic terrestrial locomotion in humans (*e.g.* Margaria, 1938) and in animals (*e.g.* Taylor et al., 1982). The advantage of the treadmill is obvious: locomotion analysis can be performed in a confined space without a long track, the velocity of progression is controlled and the subject can be connected to fixed measurement devices (oxygen consumption, electromyography, etc.). Many studies have been performed to compare treadmill locomotion with overground locomotion (*e.g.* Murray et al., 1985; Alton et al., 1998; Riley et al., 2008).

As shown by Van Ingen Schenau (Van Ingen Schenau, 1980), locomotion on the treadmill is mechanically equal to locomotion on the firm ground, as long as the belt moves at a constant speed and air drag is negligible. On the contrary, locomotion on a treadmill with an accelerating belt is not mechanically equivalent to locomotion overground while accelerating (*e.g.* Van Caekenberghe et al., 2013).

When moving on a treadmill at an average constant speed, intra-stride variations of the belt speed are observed. These intra-stride variations strongly depend on the quality of the treadmill: treadmills equipped with heavy roller and/or flywheel that have a large inertia and/or with powerful “smart” motors (i.e. with a servomechanism) minimize that effect. In good (and often expensive) treadmills, the intra-stride variations range between less than 5% at high speeds to less than 15% at low speeds (Pierrynowski et al., 1980; Riley et al., 2007; Crétual and Fusco, 2011). These intra-stride variations slightly modify the kinematics parameters (Savelberg et al., 1998). The mechanical work performed during running on the treadmill is <10% smaller than during overground running (Gosseye et al., 2010). In their paper, Gosseye et al. (Gosseye et al., 2010) proposed a method to compute the external work, *i.e.* the work necessary to move the centre of mass of the body relative to the surroundings (Cavagna, 1975), which takes the belt speed changes into account.

In the late 1980s, Kram and Powell (Kram and Powell, 1989) have mounted a force platform directly under the belt of a motorized treadmill. However, this early design was not able to measure horizontal forces accurately due to belt friction (a point made by the authors). Since the late 1990s, several laboratories have developed instrumented treadmills that measure the three components of the ground reaction force (e.g. Kram et al., 1998; Belli et al., 2001; Gottschall and Kram, 2005; Paolini et al., 2007; Gosseye et al., 2010).

Nowadays, force treadmills are commercially available in a variety of forms. However, questions raised by reviewers and/or by colleagues during congresses suggest that uncertainty still exists about the accuracy of these measures: some believe that friction between the belt and the tread-surface is summed (or subtracted) to the fore–aft component of the force applied by the feet on the belt. Consequently, the fore–aft component of the force measured by the sensors should not be equal to the force exerted by the feet on the upper surface. Others believe that on a motorized treadmill, a force is applied to the feet by the belt and that load cells placed beneath the structure will not measure this force. Therefore, part of the scientific community still believes that it is not possible to correctly measure the horizontal components of the forces exerted by the feet on the belt.

To our knowledge, no author gives a theoretical explanation of how the forces are transmitted from the upper surface of treadmill to the transducers fixed under it. In this paper, we propose a simple model of an instrumented treadmill and demonstrate theoretically that the transducers placed under the treadmill correctly measure the forces exerted by the feet on the upper side of the treadmill.

## The model of the treadmill

**Fig. 1.**
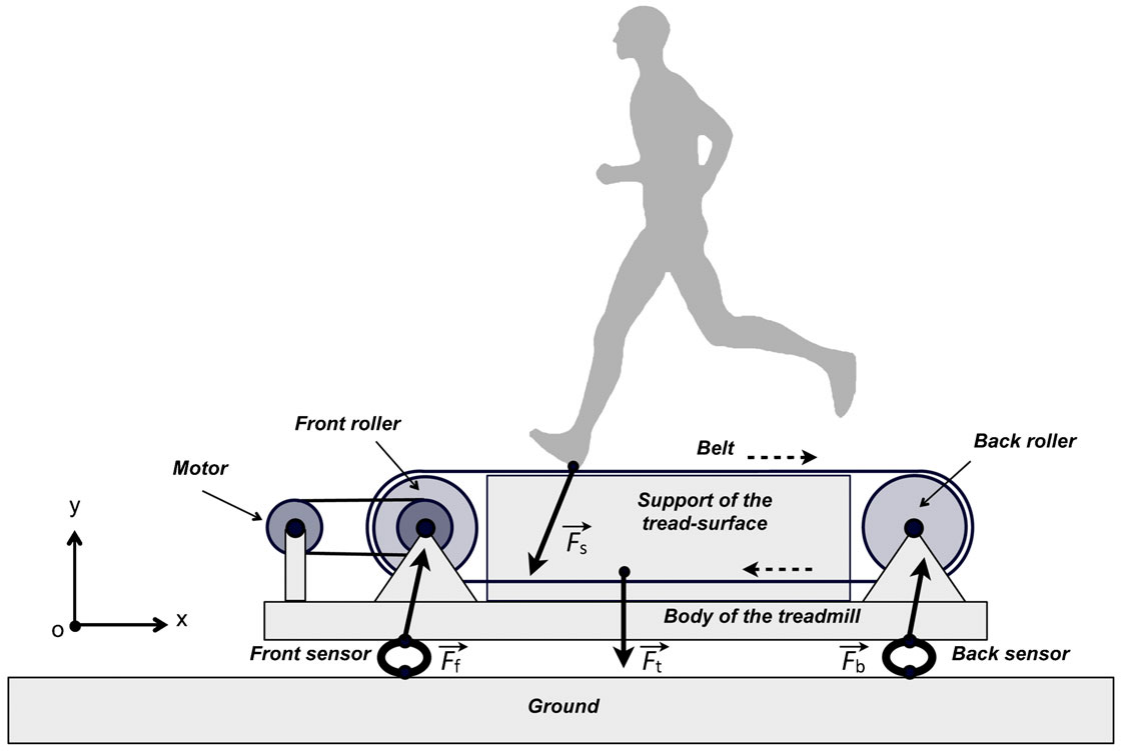
Schema of an instrumented treadmill. The interrupted arrows represent the direction of the movement of the belt. For further explanations, see text.

Fig. 1 present a schema of a subject running on a treadmill equipped with force transducers. For simplification, a projection of the treadmill on the sagittal plane is presented and only the vertical and fore–aft components of the existing forces are taken into account. Lateral component of these forces are neglected, although the same reasoning than the one presented here can be held, *mutatis mutandis*, for the lateral component of these forces.

In the schema, only the main parts of the treadmill are presented. The subject is moving on an inelastic belt supported by a tread-surface fixed on the body of the treadmill. The belt is tensioned between two rollers, which are mounted on ball bearings and attached to the body of the treadmill through a support. An electrical motor is also fixed to the body of the structure; an inelastic band is transferring motion from the motor to the front roller. The whole structure of the treadmill (*i.e.* the body, tread-surface, belt and motor) is mounted on force transducers. In this way, the transducers are the only mechanical contact between the treadmill and the external world. On a real treadmill, four transducers measuring the three components of the forces are placed close to the four corners of the treadmill. Since our model studies the forces in the sagittal plane, only two sensors are represented under the body of the treadmill, one in the front and one in the back. Each of these sensors measure the vertical and fore–aft components of the forces applied on the treadmill without any cross-talk.

## Diagram of the external forces applied on the treadmill and of the internal forces between the parts of the treadmill

Since the fixed parts of the treadmill are rigid and firmly attached to each other and since the mobile parts are moving symmetrically (Fig. 1), the acceleration and the velocity of the centre of mass of the treadmill relative to the reference frame of the laboratory are nil. According to Newton's 2nd Law, the sum of the external forces acting on the treadmill is nil:
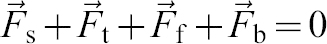
(1)where 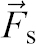 is the force exerted by the foot on the belt, 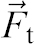 is the weight of the treadmill and 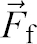 and 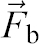 are the forces exerted by the front (subscript f) and the back (subscript b) sensors under the body of the treadmill. See Table 1 for full list of symbols used.

**Table 1.**
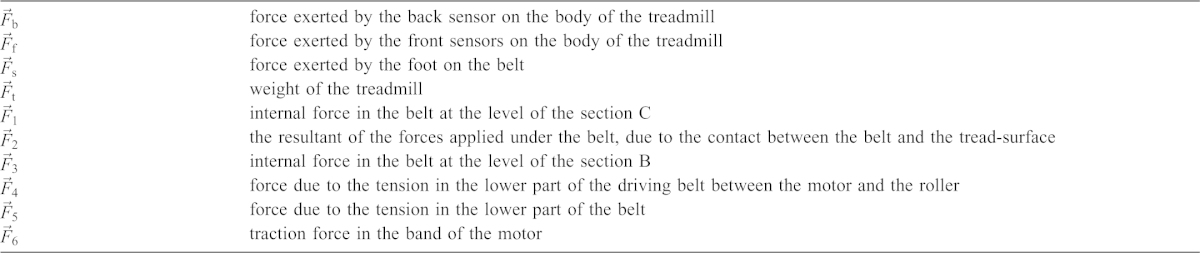
List of symbols.

In the vertical direction (y axis), since the treadmill weight does not change, the force 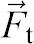 can be “removed” by changing the electrical offset of the force transducers signal output. Eqn 1 becomes:
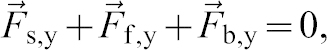
and thus:
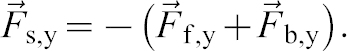
(2)In the horizontal direction (x axis), Eqn 1 becomes:
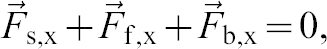
and thus:
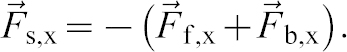
(3)Intuitively, Eqn 2 is easy to understand since no component of the treadmill is moving vertically. Eqn 3 is slightly more difficult to comprehend since an electrical motor pulls the belt and friction forces are generated at different places. One can demonstrate that all these internal forces cancel each other so that Eqn 3 is fulfilled.

**Fig. 2.**
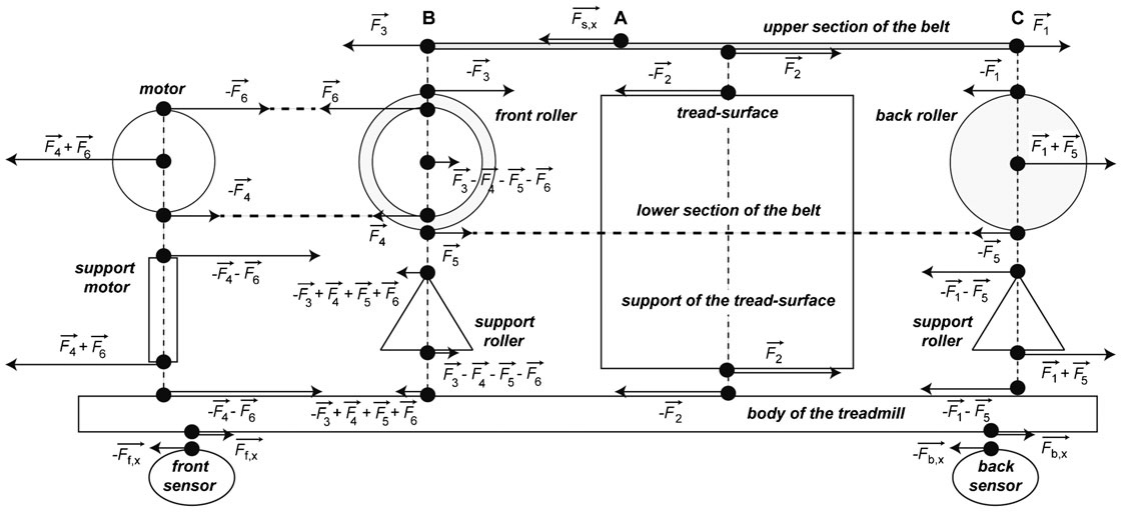
Diagram of the fore–aft components of the external forces applied on an instrumented treadmill and of the internal forces between the different components of the treadmill. For further explanations, see text.

Fig. 2 represents a schema of the horizontal component of the external and internal forces applied on the treadmill. In this diagram, each part of the treadmill is isolated and interactions with the other parts are introduced.

### 

#### Horizontal forces acting on the upper section of the belt

If the upper section of the belt is isolated between point B and C, the forces acting on it are:

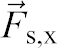
, the force applied by the foot on the belt at point A;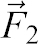, the resultant of the forces applied under the belt, due to the contact between the belt and the tread-surface;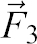, the force of traction at the front of the belt section. 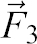 is the internal interaction between point B and the rest of the belt. 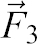 is generated by the tension of the belt between the two rollers but also by the force of the motor pulling on the belt;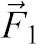, the internal force at the other end of the belt (point C).

If we suppose that the belt speed is constant, the acceleration of the centre of mass of the section B–C is nil and the equation of the forces on this section is:
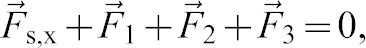
and thus:
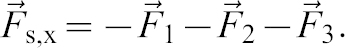
(4)

#### Horizontal forces on the front roller

The forces on the front roller are:

- the reaction force of 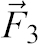, *i.e.* 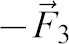;
- the traction force due to the lower part of the belt 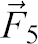 (note that 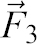 and 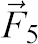 are not identical);
- the traction force 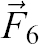 in the driving belt between the motor and the roller. Since the diameter of the roller on which the tread-belt turns and the diameter of the pulley of the motor are not equal, the magnitude of 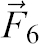 and 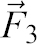 are not equal;
- the traction force 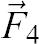 due to the lower part of the band of the motor;
- the force on the support of the roller. Since the centre of mass of the roller is fixed, this force is the resultant of the four other forces, *i.e.* 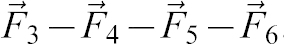.

#### Horizontal forces on the back roller

The forces on the back roller are:

- the reaction force of 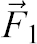, *i.e.* 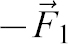;
- the reaction force of 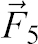, *i.e.* 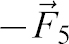;
- the force in the support of the back roller, which is the resultant of the two others, *i.e.* 
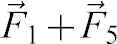
.

#### Horizontal forces on the motor

The forces on the pulley of the motor are:

- the reaction force of 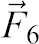, *i.e.* 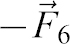;
- the reaction force of 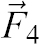, *i.e.* 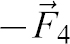;
- the force on the support of the motor, which is the resultant of the two others, *i.e.* 
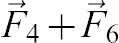
.

#### Horizontal forces on the body of the treadmill

Since the tread-surface, the supports of the rollers and of the motor are rigid; the following forces are transmitted to the body of the treadmill:

- at the level of the tread-surface, the reaction force of 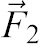 is 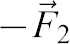;
- at the level of the front roller, the reaction force of 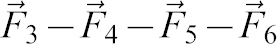 is 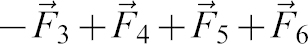;
- at the level of the back roller, the reaction force of 
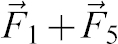
 is 
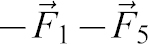
;
- at the level of the motor, the reaction force of 
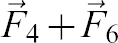
 is 
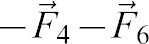
.

Two other forces are applied to the body of the treadmill by the front and back sensors: 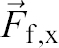 and 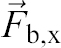. The equation of the forces applied on the body is:
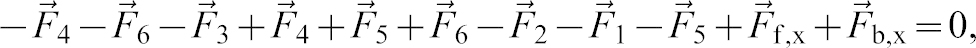
which becomes:
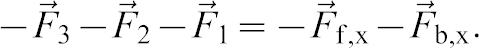
(5)Since 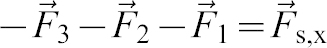 Eqn 4, Eqn 5 becomes:
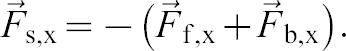
(6)Eqn 6 (equal to Eqn 3) shows that the force applied horizontally on the belt by the feet of the subject is equal to the sum of the forces measured on the two sensors.

## Discussion

Our simplified model of the treadmill shows by a theoretical example that transducers placed under the body of the treadmill correctly measure the external forces exerted by the feet on the belt. Even in more sophisticated models, like dual belt treadmills or treadmill designed for running, wheel chair training, skating or skiing (using wheeled skate or ski), all internal forces between the parts of the treadmill cancel each other, as long as the whole structure of the treadmill is mounted on the force transducers.

Nevertheless, our simplified model hides some measurement errors that occur on a real treadmill. The main problem is due to the mechanical vibrations induced by the treadmill on the sensors. Indeed, due to small unbalances in the rollers and in the mechanical parts of the motor, due to a lack in rigidity of the structure, vibrations are generated and create small oscillations of the centre of mass of the treadmill, which in turn induce noise on the force measurements. The noise disappears when averaging or integrating forces over several steps but it can affect the measurement of peak forces. These vibrations reduce also the signal-to-noise ratio, and thus the precision, in the calculation of the centre of pressure of the ground reaction force (Winter, 2009). Other measurement errors can also occur due to a tread-surface deflection, which will store and release mechanical energy during contact. A too low natural frequency of the treadmill may also induce resonant frequencies of the treadmill. This occurs if the frequencies generated by the ground reaction force matches the treadmill natural frequency of vibration.

In a well-designed treadmill (*i.e.* a treadmill sufficiently rigid but not too heavy), the frequency content of the mechanical noise is higher than the frequency content of the force exerted by the feet on the belt (Kram et al., 1998). In this case, the noise can be removed by low-pass digital filtering.

Our theoretical demonstration also confirms the experimental observations of Kram et al. (Kram et al., 1998). These authors compared the forces obtained on their instrumented treadmill with data obtained from a force platform runway. Their results indicate that the ground-reaction forces measured in the two situations were similar (see their figure 4).

## Conclusion

The treadmill is a well-known and useful tool for athlete training, clinical rehabilitation or recreational exercising. Here we show that it can also be used as an objective evaluation tool to assess the ground reaction forces during multiple strides of non-accelerating locomotion.
